# Sorafenib treatment during partial hepatectomy reduces tumorgenesis in an inflammation-associated liver cancer model

**DOI:** 10.18632/oncotarget.6638

**Published:** 2015-12-17

**Authors:** Tamar Zahavi, Tali Lanton, Mali Salmon Divon, Asher Salmon, Tamar Peretz, Eithan Galun, Jonathan H. Axelrod, Amir Sonnenblick

**Affiliations:** ^1^ Sharett Institute of Oncology, Hadassah-Hebrew University Medical Center, Jerusalem, Israel; ^2^ Goldyne Savad Institute of Gene Therapy, Hadassah-Hebrew University Medical Center, Jerusalem, Israel; ^3^ Department of Molecular Biology, Ariel University, Ariel, Israel

**Keywords:** Sorafenib, hepatectomy, liver cancer, stellate cells, Mdr2 knockout

## Abstract

The long-term prognosis after resection of hepatocellular carcinoma (HCC), which is one of the treatment options for early-stage HCC, remains unsatisfactory as a result of a high incidence of disease recurrence. Recent studies performed in murine models revealed a link between liver regeneration under chronic inflammation and hepatic tumorigenesis. Sorafenib is a potent drug for advanced HCC with multikinase inhibition activity. We propose that inhibition of signal transduction pathways which are activated during hepatectomy, using Sorafenib, will reduce accelerated tumorigenesis. To test this hypothesis, we studied the Mdr2-knockout (KO) mouse strain, a model of inflammation-associated cancer, which underwent partial hepatectomy (PHx) at three months of age, with or without Sorafenib.

Here we show that Sorafenib treatment during PHx inhibited different signal transduction pathways at the multikinase levels, but did not result in increased morbidity or mortality. At the early stages after PHx, Sorafenib treatment had no effect on the course of proliferation, apoptosis and DNA repair in the regenerating liver, but resulted in decreased stellate cells activation and inflammatory response. Finally, we show that Sorafenib treatment during PHx at three months of age resulted in decreased fibrosis and tumor formation at 8.5 months.

In conclusion our study indicates that short-term Sorafenib treatment during PHx is safe and effective in inhibiting inflammation-associated cancer, and is therefore a potential strategy for recurrence prevention in patients with early-stage HCC treated with PHx.

## INTRODUCTION

Hepatocellular carcinoma (HCC), one of the leading causes of cancer mortality worldwide, commonly develops in an inflamed liver following a prolonged chronic hepatitis state [1, 2]. Partial liver resection (partial hepatectomy; PHx) is one of the treatment options for early-stage HCC patients [3–6]. However, survival rates following PHx are suboptimal, mostly due to tumor recurrence, which within five years is in the range of 75 to 100% of cases [7–10]. It is estimated that 60 to 70% of recurrences are attributed to intrahepatic lesions undetected by the time of resection, whereas 30 to 40% are de novo HCCs [11–14].

HCC in mice and humans share common features and various mouse models of this disease have been studied to uncover the molecular mechanisms of liver cancer [15]. Animal studies investigating the effects of liver regeneration on tumor progression were performed using transplanted tumor cells, or using chemically induced tumors. In these animal models, PHx has been shown to affect and enhance both the initiation and promotion phases of hepatocarcinogenesis, when compared to sham operation [16–18]. We selected the Mdr2-knockout (KO) mouse as a model due to its similarities to human HCC development and due to a study that revealed that PHx led to enhanced hepato-carcinogenesis in this model [19–21].

Sorafenib (Nexavar®, Bayer HealthCare Pharmaceuticals) is a small molecule that inhibits tumor-cell proliferation and tumor angiogenesis by inhibiting the serine–threonine kinases BRAF and the receptor tyrosine kinase activity of vascular endothelial growth factor receptors (VEGFRs) 1, 2, and 3 as well as the platelet-derived growth factor receptor β (PDGFR-β) [22]. Cellular signaling that is mediated by the BRAF, VEGF and PDGFR-β pathways has been implicated in the molecular pathogenesis of hepatocellular carcinoma, providing a rationale for investigating Sorafenib for this indication [15]. Indeed two phase 3 studies have shown that Sorafenib prolonged median survival and the time to progression by nearly three months in patients with advanced HCC [23, 24].

Since VEGFR, PDGFR and BRAF are key regulators of liver regeneration [25] and are essential for promoting inflammation-associated cancer [15], we hypothesized that inhibition of these signal transduction pathways during PHx using Sorafenib would reduce accelerated tumorigenesis.

## RESULTS

### Short-term Sorafenib treatment during PHx inhibits different signaling pathways in the chronic inflamed liver

**Figure 1:**
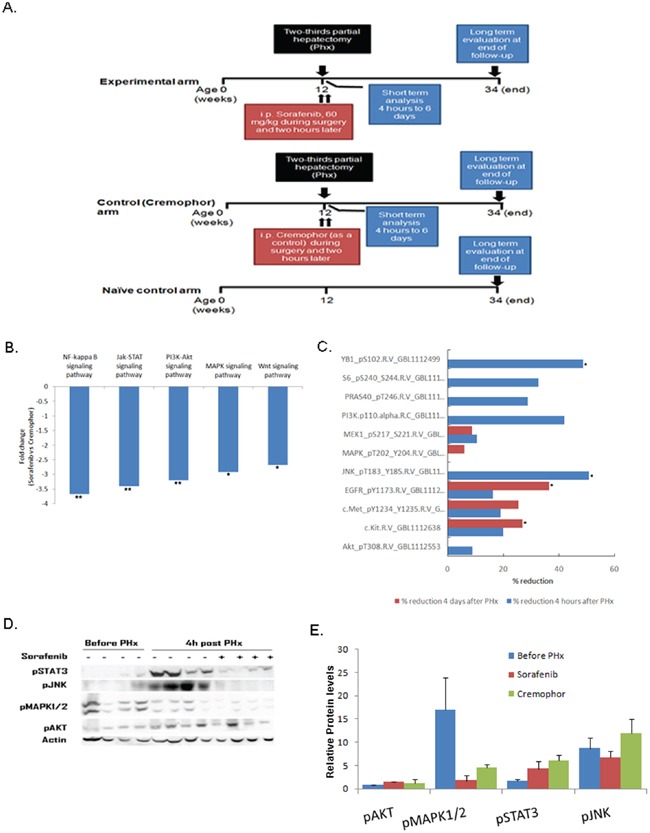
Short-term Sorafenib treatment during PHx inhibits different signaling pathways in the chronic inflamed liver Mdr2- KO mice were subjected to PHx at three months of age and treated with Sorafenib or Cremophor immediately and two hours following surgery. **A.** Experimental schedule **B.** Down-regulated intracellular signaling pathways in livers of short-term Sorafenib treated mice four hours post PHx compared to controls. RNA from short-term Sorafenib or Cremophor treated mice four hours post PHx was analyzed using RNA sequencing. The graph shows the fold reduction of signaling pathways obtained by short-term Sorafenib treatment compared to the values in the control Cremophor-treated mice. **C.** Total hepatic proteins from short-term Sorafenib or Cremophor treated mice four hours and four days post PHx were analyzed using reverse phase protein array (RPPA). The graph shows the percentage reduction of signaling proteins obtained by short-term Sorafenib treatment. The data are presented as percentage decrease compared with the values in control Cremophor-treated mice. Four mice of each group were subjected to RPPA analysis with 166 antibodies. **D**–**E** Western blot analysis (D) and quantification (E) of total hepatic protein levels of phosphorylated (p)STAT3, phosphorylated (p)JNK, phosphorylated (p) MAPK1/2, and phosphorylated (p)AKT from mice before and four hours following PHx. Each band represents one single mouse sample in the indicated group. For all experiments n=4-5 per time point per group: **P < 0.01; *P < 0.05.

Although it is known that Sorafenib blocks various intracellular signaling pathways in the liver [26, 27], analysis of the signaling molecules affected by Sorafenib treatment in the chronic inflamed liver model during PHx has not been determined. To address this question, we performed 70% PHx on three-month-old (inflamed liver) Mdr2- KO mice that were treated with Sorafenib or Cremophor (as a control) immediately and two hours after surgery (Figure 1A). Gene expression and proteomic analysis revealed that short-term Sorafenib strongly inhibited directly or indirectly various intracellular signaling pathways (Figure 1B–1E). First, we used the RNA-seq technology in order to compare the gene expression profile of livers from short-term Sorafenib treated mice four hours post PHx to controls. Gene set enrichment analysis using the GAGE method [28] revealed that four hours after PHx, Sorafenib significantly reduced various intracellular signaling pathways such as MAPK, Jak-STAT, PI3-AKT, NF-kB and Wnt (Figure 1B). At the protein level, we interrogated proteins and phosphoprotein profiles associated with short-term Sorafenib treatment during PHx by reverse-phase protein array (RPPA) analysis (n=4-5 mice per time point per group). Using this analysis, four hours and four days following surgery, we detected that Sorafenib inhibited the activation of key signaling players such as of PI3K/AKT, RAS/EGFR/MEK, c-kit/met and JNK (Figure 1C and Supplementary Figure S1). To validate these observations, the phosphorylation levels of selected proteins were measured by Western blot at four hours following PHx in liver tissues from short-term Sorafenib treated mice (n=4 mice per group). As shown in Figure 1D–1E, hepatic p-STAT3, p-JNK, p-MAPK1/2 and p-AKT levels were reduced in the Sorafenib treated mice in comparison to control mice.

### Short-term Sorafenib treatment during PHx does not affect the cellular response of hepatocytes in the chronic inflamed liver

**Figure 2:**
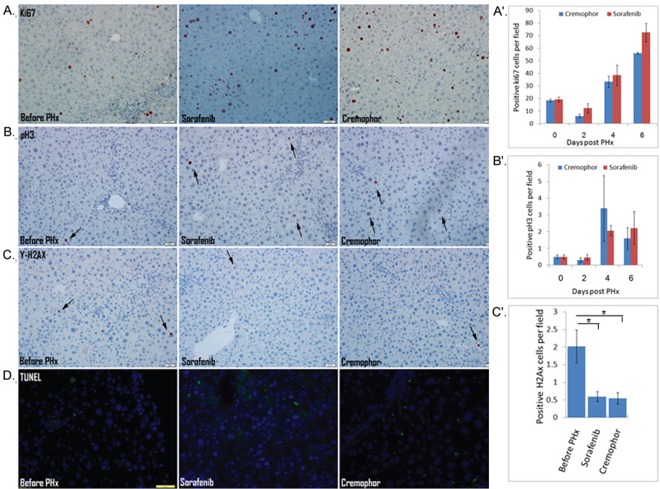
The cellular response of hepatocytes is not directly affected by short-term Sorafenib treatment during PHx **A–C** IHC staining for Ki67-positive cells (A) and PH3-positive cells (B) in mice before and on day 4 post PHx, and for γ-H2AX-positive cells in mice before and on day 2 post PHx **C.** (A′-C′) quantification of Ki67-positive cells (A′), PH3-positive cells (B′) at the indicated time points, and γ-H2AX-positive cells (C′) on day 2 following PHx in liver tissue sections of mice for 10 randomly selected fields. **D.** IHC staining for apoptosis by TUNEL assay in mice before and on day 2 following PHx. (DAPI, blue; apoptosis, green). For all experiments: n=4-5 per time point per group, *P < 0.05.

Because cell signaling governs basic cellular activities such as proliferation, apoptosis and DNA repair, and short-term Sorafenib treatment during PHx inhibits signaling pathways, one would expect that short-term Sorafenib during PHx may have a direct effect on the hepatocyte cell cycle and DNA repair response. We therefore subjected three-month-old Mdr2-KO mice to PHx and analyzed the cellular response of the hepatocytes using immunohistochemical markers in mice treated with Sorafenib during PHx. Surprisingly, the cellular response was not significantly affected by short-term Sorafenib treatment in comparison to controls (Figure 2). First, we evaluated hepatocyte proliferation kinetics using immunostaining with Ki67 (general cell cycle marker) and phosphorylated H3 (PH3) (marker of G2/M phase at cell cycle) at several time points (days 2, 4 and 6) post-PHx. The results indicated that in Sorafenib treated mice, there were no statistically significant differences in the general cell cycle marker ki67 or the specific PH3 marker, as compared to control-Cremophor- treated mice (Figure 2; A,A′,B,B′). Since Mdr2-KO mice have a high incidence of DNA damage which induces activation of the DNA damage-response pathway [21], we examined the presence of DNA damage in livers of short-term Sorafenib treated mice two days post PHx using the phosphorylation of γ-H2AX as a marker of double strand breaks (DSBs) [29]. In both groups, we observed that the control mice and the Sorafenib-treated mice hardly had any γ-H2AX-labeled hepatocytes (Figure 2C, C′). Thus, our results suggest that short-term Sorafenib does not affect the presence of DSBs in hepatocytes. Next, we examined the presence of apoptosis in livers from short-term Sorafenib treated mice, two days post PHx. There was no significant TUNEL staining in the short-term Sorafenib treated mice or the control Cremophor-treated mice (Figure 2D). In conclusion, our results suggest that following PHx, the cellular response of hepatocytes is not directly affected by short-term Sorafenib treatment.

### Short-term Sorafenib treatment during PHx resulted in a decreased inflammatory response

**Figure 3:**
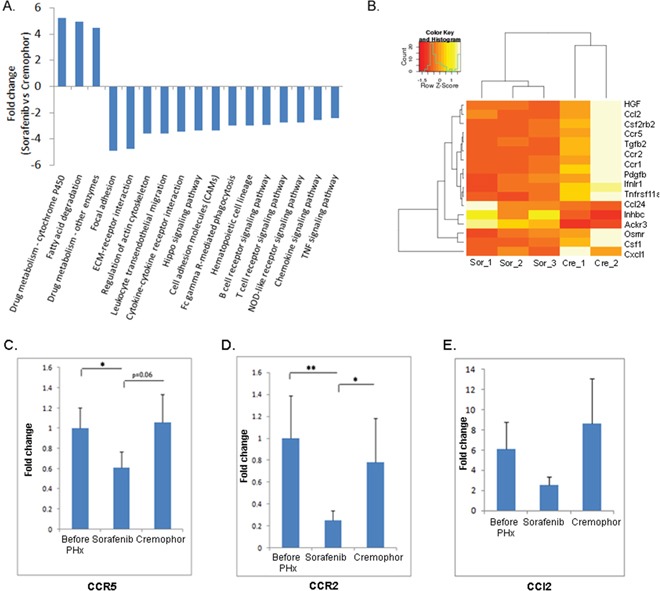
Short-term Sorafenib treatment during PHx resulted in decreased inflammatory signaling pathways **A.** RNA from short-term Sorafenib or Cremophor treated mice four hours post PHx was analyzed using RNA sequencing. A marked reduction of many inflammatory signaling pathways in livers of short-term Sorafenib treated mice four hours post PHx compared to controls. For all pathways the FDR is ≤ 0.05 **B.** Heatmap of the inflammatory chemokine genes, which almost all of them are down-regulated in short-term Sorafenib treated mice, but not in short-term Cremophor-treated mice four hours post PHx (n=2-3/group). **C–E** Expression of (C) CCR5 (D) CCR2 and (E) CCl2 in liver extracts of short-term Sorafenib or Cremophor treated three- month-old mice, as determined by real-time PCR before and four hours following PHx (n=6-8/group). For all experiments: **P < 0.01; *P < 0.05.

As noted above, in an effort to understand the molecular pathways that are affected by short-term Sorafenib treatment during PHx in livers of Mdr2-KO mice, we performed a gene expression profiling study in Sorafenib treated mice four hours post PHx. Gene set enrichment analysis using the GAGE method [28] revealed a marked reduction of many inflammatory signaling, such as leukocyte migration, TNF signaling, chemokine signaling, etc. (Figure 3A). We performed a hierarchical clustering between the expression of the inflammatory chemokines in the livers of short-term Sorafenib treated mice and the control-Cremophor treated mice. This investigation revealed a marked reduction of inflammatory chemokines such as CCR5, CCR2 and CCl2 in short-term Sorafenib treated mice (Figure 3B). Using real-time PCR, we confirmed that the expression of CCR5, CCR2, and CCl2 was indeed down-regulated in the livers of short-term Sorafenib treated mice, compared to control-Cremophor treated mice (Figure 3C–3E). Thus, short-term Sorafenib treatment during PHx is associated with decreased chemokine expression patterns.

**Figure 4:**
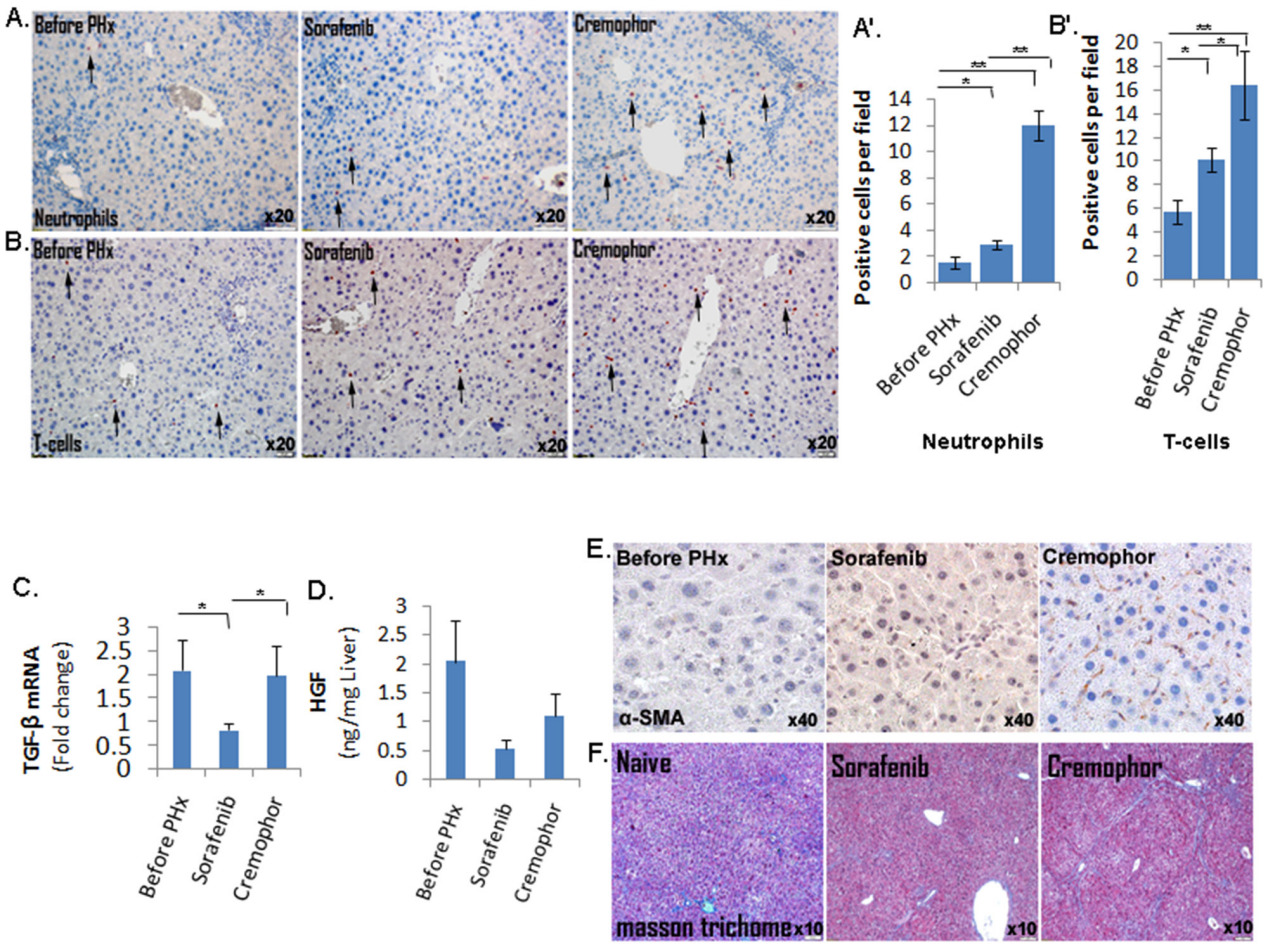
Short-term Sorafenib treatment during PHx resulted in a decreased inflammatory response and stellate cells activation **A.** Gr-1 IHC staining of neutrophils in mice before and four hours following PHx (n=5-6/group). **B.** CD3 IHC staining of CD3 T-cells in mice before and on day 4 following PHx (n=4/group). Quantification of the Gr-1- and CD3-positive cells (A′ and B′, respectively) was done at the indicated time points for 10 randomly selected fields. **C.** Expression of TGF-β in liver extracts of three- month-old mice, as determined by real-time PCR before and four hours following PHx (n=7-8/group). **D.** HGF levels in the livers of three- month-old mice determined by ELISA assay before and four hours following PHx (n= 3/group). **E.** Immunohistochemistry staining of α -SMA in liver tissue sections of mice before and on day 2 following PHx (n=4-5/group). For all experiments: **P < 0.01; *P < 0.05. **F.** Assessment of extracellular collagen deposits indicated by Masson trichome staining at 8.5 months (n =6-7/group).

Next, we evaluated whether the cellular inflammatory response related to inflammatory chemokines was also down-regulated in the livers from short-term Sorafenib treated mice post PHx. Indeed, immunohistochemistry analyses showed that in the short-term Sorafenib treated mice, the numbers of infiltrating neutrophils (Gr1+ cells) and CD3 T-cells post PHx were reduced compared to those of control-Cremophor treated mice (Fig. 4A, 4B). These observations were supported by the RPPA analysis which showed a decreased expression of the lymphocyte-specific protein tyrosine kinase (Lck) protein which is essential for T-lymphocyte activation and differentiation [30] in the Sorafenib treated mice four days post PHx (Supplementary Figure S1B). Surprisingly, despite the decline in the inflammatory chemokine receptors CCR5 and CCR2, the numbers of F4/80+ macrophages were not decreased in the livers of short-term Sorafenib treated mice four hours post PHx (Supplementary Figure S2).

### Short-term Sorafenib treatment during PHx resulted in decreased stellate cells activation and fibrosis

Our gene expression profiling study demonstrated downregulation of the TGF-β and hepatocyte growth factor (HGF) in Sorafenib treated mice four hours post PHx (Figure 3B). These observations were confirmed using real-time PCR which showed that mRNA expression of TGF-β was significantly lower in the livers of short-term Sorafenib treated mice four hours post PHx, compared to control mice (Figure 4C); in addition, using the ELISA assay we demonstrated lower HGF levels in livers of short-term Sorafenib treatment mice two days post PHx, compared to Cremophor- treated control mice (Figure 4D). Since both TGF-β and HGF are mainly produced by hepatic stellate cells (HSCs) in the regenerating liver [25], we hypothesized that Sorafenib treatment during PHx may affect HSC function. Indeed, the expression of α -SMA, a marker for HSC activation, was much lower in the Sorafenib treated mice 48 hours post PHx, compared to Cremophor- treated control mice (Figure 4E).

Since HSCs produce most of the extracellular deposits and matrix metalloproteinases (MMPs) involved in fibrogenesis (30), we decided to test whether inhibition of HSC function and downregulation of TGF-β (a major pro-fibrotic factor) by Sorafenib during PHx at the age of three months, would result in reduced fibrosis at later stages. As shown in Figure 4F, mice that were subjected to PHx at three months of age and treated with short-term Sorafenib had significantly lower collagen deposits and fibrosis in their livers than control mice at the age of 8.5 months, as shown by the Masson trichome staining.

### Less tumorgenesis in short-term Sorafenib treated mice

**Figure 5:**
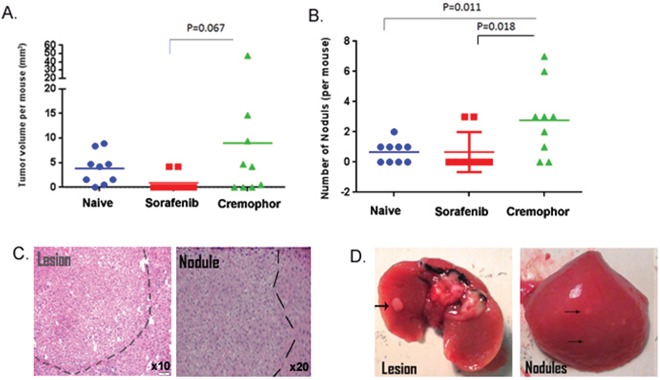
Short-term Sorafenib treatment during PHx reduces tumorigenesis in the Mdr2-KO model Mdr2-KO mice were subjected to PHx at three months of age and treated with Sorafenib or Cremophor immediately and two hours following the surgery. Tumors were measured and counted in naïve (no PHx), short-term Sorafenib (during PHx at three months) and short-term Cremophor (during Phx at three months) treated mice at 8.5 months of age. **A.** Total tumor volume per mouse was calculated from all tumors larger than or equal to 2 mm (n=9/group). **B.** Number of dysplastic nodules smaller than or equal to 1 mm per mouse. (n=9/group). **C.** Representative H&E liver sections revealing internal tumors and dysplastic nodules in short-term Cremophor treated mice. **D.** Representative images of harvested livers and tumors from short-term Cremophor treated mice.

Here, we showed that short-term Sorafenib treatment during PHx resulted in less fibrosis and inflammatory response. In humans and mice, fibrosis and inflammation are believed to be a prerequisite for HCC development [2, 31]. To test the effect of short-term Sorafenib treatment during PHx on tumor development, Mdr2-KO mice were subjected to PHx at three months of age and treated with IP injections of Sorafenib or Cremophor immediately and two hours following surgery. Mice were then followed for five months, sacrificed at the age of 8.5 months and analyzed for the presence of visible liver tumors. This analysis revealed that both the average tumor lesion volume and the number of lesions per mouse were reduced in the Sorafenib treated group, compared to the Cremophor-control mice (Figure 5A–5B). Moreover, the PHx induced accelerated tumorigenesis phenomenon [21], which was demonstrated in the Cremophor-control mice in comparison to the naïve control arms (that were not subjected to PHx), was abolished in the Sorafenib treated arm (Figure 5A–5B). Hematoxylin and Eosin (H&E) staining of livers from 8.5-month-old mice confirmed the macroscopically results. In livers of short-term Sorafenib treated mice that had barely macroscopically detected lesions, there was a tumor-clear profile and few nodules, while the livers of Cremophor-control mice had tumor profiles and nodules (Fig. 5C–5D).

Finally, we evaluated the safety aspects of our approach (Table 1). Hepatocyte damage was evaluated by measuring serum ALT. Liver enzyme levels of 8.5-month-old mice were measured to assess liver damage. High levels of ALT with no significant differences were detected in the serum of all mouse groups, implying that the hepatocyte damage in all mouse groups was significant with no detrimental effect of Sorafenib. Survival rates and liver/body weight at 8.5 months of age were not significantly different in all three mouse groups (Table 1). Overall, our results indicate that short-term Sorafenib treatment during PHx is safe and effective in inhibiting inflammation-associated cancer.

**Table 1:** Evaluation of safety aspects

	Naïve group	Sorafenib group	Control (Cremophor) group
Liver/Body weight per mouse (grams)	8.48±0.4	7.89±0.85	9.6±1.85
ALT (enzyme units)	641±228	534±209	752±278
Survival of mice at 8.5 months	9/10	9/10	9/10

Liver damage was assessed by measuring the liver enzyme ALT level in the serum. Hepatocyte damage, survival rates and liver/body weight at 8.5 months of age were not significantly different in all three mouse groups (n = 9/group).

## DISCUSSION

In this study, we demonstrated that Sorafenib treatment during PHx is safe and effective in inhibiting the development of inflammation-associated cancer in the Mdr2-KO model.

The concept that accelerated tumor progression due to PHx can be suppressed using Sorafenib without compromising liver regeneration was previously demonstrated with transplanted tumor cells and orthotropic HCC models. In these studies, it was suggested that the postoperative activation of the RAF-MEK-ERK signal transduction pathway sensitizes HCC to Sorafenib [26, 27]. However, these models lack underlying liver inflammation as in the case of most humans with HCC and the effect of Sorafenib on the transplanted tumor was direct.

In order to overcome these limitations, we used the Mdr2-KO mouse strain. These mice lack the Mdr2 P-glycoprotein, which is responsible for the phosphatidylcholine transport across the canalicular membrane. The absence of phospholipids from bile leads to portal inflammation and a slowly developing HCC, which closely mimics the human disease in this regard [19, 20]. Therefore we were able to explore the mechanisms by which the inflammatory microenvironment affects liver regeneration and the effect of both, inflammation and regeneration on hepatocarcinogenesis in the context of Sorafenib treatment during PHx. Moreover, we used a very constrained time frame and provided Sorafenib in two doses, during and two hours after PHx, which enabled us to test the effect during the post PHx period.

Indeed, we uncovered a novel mechanism by which Sorafenib inhibits stellate cells activation post PHx. This inhibition was accompanied by a reduced expression of HGF and TGFβ, lower chemotaxis and inflammatory response in the short term and reduced fibrosis in the long term. Our observations support recent studies showing that Sorafenib may induce the suppression of collagen accumulation and growth of stellate cells through downregulation of HGF and TGFβ [32–34]. In our study, Sorafenib treatment had no significant effect on the course of proliferation, apoptosis and DNA repair in the regenerating liver. The opposing effects of HGF and TGFβ on hepatocyte proliferation [25] may explain why no changes were seen in hepatocyte proliferation.

Finally, our data show that short term Sorafenib treatment during PHx at three months resulted in a decreased tumor formation at 8.5 months. Our study thus indicates that short-term Sorafenib treatment during PHx is safe and effective in inhibiting inflammation-associated cancer through an indirect stromal inhibition, and is therefore a potential strategy for recurrence prevention in patients with early stage HCC treated with PHx. Of note, adjuvant Sorafenib has been tested in the STORM trial with a clearly negative outcome[35], however the inclusion criteria for the trial required at least three weeks from resection which might be too late to achieve the desirable effect around the hepatectomy period as demonstrated in our model. Despite the risks for the frail patients with HCC and liver inflammation, a cautious evaluation of Sorafenib treatment during PHx in humans may be found to be useful.

## MATERIALS AND METHODS

### Mice

Mdr2-KO mice (Fvb/NJ background) were bred in-house from breeding pairs originally purchased from the Jackson Laboratory (Bar Harbor, ME). Animals were maintained in a climate-controlled environment at 23°C, exposed to a 12 : 12 h light : dark cycle, fed standard laboratory chow, and given water *ad libitum*, under SPF conditions as assessed by regular microbiological screening. Animal experiments were performed according to a protocol approved by the Animal Care Committee of the Hebrew University (Jerusalem, Israel).

### Partial hepatectomy and drug treatment

Two-thirds partial hepatectomy (PHx) was performed under ketamine and xylazine anesthesia and consisted of midline laparotomy with separate ligation and removal of the left and anterior median lobes, as described previously [36]. At indicated time points after surgery, mice were sacrificed by isoflurane^®^ inhalation, upon which livers were harvested and blood samples taken (Figure 1A). Liver specimens were either fixed in 4% buffered formalin or snap-frozen in liquid nitrogen for further analysis. Sorafenib, 60 mg/kg, or its vehicle (Cremophor/ethanol/sterile saline) was administered by intraperitoneal (i.p.) injection during surgery and two hours later. The dosing volume used was 0.12 mL/25g body weight. The proportions of cremophor/ethanol/sterile saline were 12.5% Cremophor, 12.5% ethanol, and 75% sterile saline. For the animals receiving Sorafenib, the drug was first dissolved in a 50% Cremophor /50% ethanol mixture and saline was then added to reach the final volume immediately prior to application. Animals treated with the vehicle only as controls, received the analogue fluid mixture without the drug. Cremophor EL was purchased from Sigma (Sigma-Aldrich).

### Blood sample analysis

Blood samples were collected using cardiac puncture. Levels of the liver enzyme alanine aminotransferase (ALT) in sera were measured with Reflotron^®^ (Roche, Mannheim, Germany).

### Western blotting analysis

Protein extracts were prepared from tissue samples (∼50 mg) by homogenization in 500 μl whole cell lysis buffer (1% NP-40, 10 mM Tris pH 7.8, 150 mM NaCl, 40 mM EDTA, 10 mM Na-Pyrophosphate, 10 mM NaF, 1mM PMSF, 4 mM Orthovanadate, Minicomplete iprotease inhibitor^®^). Protein extracts (30 μg) were separated by polyacrylamide gel electrophoresis. Blottings were incubated one hour at RT in a blocking buffer containing 5% skim milk and then incubated overnight at 4°C with mouse monoclonal anti-phosphorylated STAT3 (Santa Cruz), rabbit monoclonal anti phosphorylated AKT (Ser473) (Cell Signaling, Danvers, MA), mouse monoclonal anti Phospho-p44/42 MAPK (Erk1/2) (Thr202/Tyr204) (Cell Signaling, Danvers, MA), mouse monoclonal anti Diphosphorylated-JNK (JNK-PT48) (Sigma) and beta-actin mouse monoclonal antibody (Sigma-Aldrich), and subsequently, with peroxidase-conjugated goat anti-mouse or anti-rabbit immunoglobulin G (Dako) for one hour at room temperature.

### Immunohistochemistry

For histological analysis, liver tissue was cut into 5-mm sections, deparaffinized with xylene, and hydrated through graded ethanol. Ki67 was stained using Rabbit monoclonal anti-Ki67 antibody (Thermo Scientific) and diluted 1:100; F4/80 was stained using rat monoclonal anti-F4/80 antibody (MCA497; Serotec, Raleigh, NC) diluted 1:200. Neutrophils were stained using Rat anti- Mouse Gr-1 antibody (MCA2387, Serotec) diluted 1:200; γ-H2AX was stained using mouse antibody to phospho-H2AX diluted 1:100 (05-636; Upstate). Rat anti Mouse CD3 antibody diluted 1:300 (MCA500G, Serotec). Rabbit anti-phospho-Histone H3 (Ser10) antibody diluted 1:600 (06-570, Upstate). α -Smooth muscle antibody diluted 1:300 (A2547, Sigma Aldrich). For all staining, we used a conjugated horseradish peroxidase secondary Ab (anti-mouse and -rabbit [Envision; Dako] and anti-rat [Histifine; Nichirei, Osaka, Japan]) for 1 hour and developed it with AEC for 15 minutes. TUNEL staining was performed with an in situ Cell Death Detection Kit (Roche Diagnostics). H&E and Masson trichrome staining were performed according to accepted protocols. For the quantitative assessment of F4/80 staining, we used the Ariol system (Genetix USA Inc., San Jose, CA) for automated cell image capture and analysis.

### Reverse phase protein array (RPPA) analysis

Reverse phase protein array (RPPA) analysis was performed by the Functional Proteomics Core Facility at MD Anderson Cancer Center. Cellular proteins were denatured by 1%(w/v) SDS in the presence of β-mercaptoethanol and adjusted to a final concentration of 6 μg/μL. Samples were diluted in five serial 2-fold dilutions in a dilution buffer (lysis buffer containing 1% SDS) and arrayed on nitrocellulose-coated slides. Each slide was probed with antibodies by the tyramide amplification approach and visualized by DAB colori- metric reaction. Slides were scanned, analyzed and quantified using the Array-Pro Analyser software to generate spot intensity. Relative protein levels for each sample were determined by interpolation of each dilution curve from the ‘standard curve’ (supercurve) of the slide (antibody). The protein concentrations of each set of slides were then normalized for protein loading and antibody variation adjustment and the linear values were transformed to median-centred Log2 values which were used for downstream analysis. Differential protein expression analysis was performed using the R package limma from the Bioconductor framework by applying the moderated t statistics. Heatmap dendrograms were generated using Euclidean distance and the complete linkage method.

### RNA extraction and real-time PCR

Total RNA was extracted from livers of 3-month-old mice using TRIzol reagent (Invitrogen Life Technologies, Carlsbad, CA), according to the protocol recommended by the manufacturer. Complementary DNA (cDNA) was obtained by reverse transcription of 850 ng of total RNA in a final reaction volume of 20 μL containing 4 μL qScript Reaction Mix and 1 μL qScript Reverse Transcriptase (Quanta BioSience). Quantitative real-time PCR assays, containing the primers and probe mix for transforming growth factor beta (TGF-β2), CCR5, CCR2, and CCl2, were purchased from Biosearch Technologies and utilized according to the manufacturer's instructions. PCR reactions were carried out in a final reaction volume of 10 μL containing 30 ng of cDNA template, 5 μL of PerfeCta SYBER Green FastMix, ROX (Quanta Biosience) and 1μL of primers mix. All reactions were run in triplicate, and the housekeeping gene, HPRT (Biosearch Technologies), was amplified in a parallel reaction for normalization.

### Gene expression

Total RNA was sent for sequencing using the Illumina TruSeq protocol, on the HiSeq 2500 sequencing machine. Quality control checks on the raw sequence data were done using the FastQC tool [.http://www.bioinformatics.babraham.ac.uk/projects/fastqc/]. Then the Trim_galore [http://www.bioinformatics.babraham.ac.uk/projects/trim_galore/] tool which is based on CutAdapt was used for adapter trimming, and removing low quality bases from the ends of reads. Clean reads were mapped to the mouse genome (mm10) using tophat2 [37]. Next, the number of reads mapping each mouse gene (as annotated in Ensembl release 74) was counted using the ‘union’ mode of HTseq-count script [38]. Differential expression analysis was performed using the edgeR package from the Bioconductor framework [39]. Briefly, features with less than 1 read per million in 3 samples were removed. The remaining gene counts were normalized using the TMM method, and the exact negative binomial test was used to find differentially expressed genes. Only genes with a false discovery rate (FDR) value of less than 0.05 were considered as differentially expressed. Gene set enrichment and pathway analysis was done using the GAGE method [28].

### Enzyme-Linked immunosorbent assay

Liver protein lysates were diluted (1:2) in Calibrator Diluent (from an MHG00 kit), and chemokine levels were determined by the enzyme-linked immunosorbent assay (ELISA) using a mouse-HGF kit (kit MHG00; R&D Systems, Minneapolis, MN), according to the manufacturer's specifications.

### Visual determination of tumor development

Tumor load was calculated by counting the number of visible surface tumors larger than or equal to 0.2 mm in each mouse liver. Tumor volume was calculated according to the equation for sphere volume (V=4/3 πr3), where V = volume, r = tumor radius. The number of nodules was calculated by counting the number of visible surface nodules smaller than or equal to 0.1 mm in each mouse liver. The student paired two-tailed *t* test was used to compare groups. Values of p ≤ 0.05 were considered significant.

## SUPPLEMENTARY FIGURES


