# Supplementary material for: Sorafenib treatment during partial hepatectomy reduces tumorgenesis in an inflammation-associated liver cancer model
Source: Oncotarget. 2015 Dec 17;7(4):4860–70. doi: 10.18632/oncotarget.6638 (PMC4826248; doi:10.18632/oncotarget.6638)
Supplement: Supplementary file 1 [file oncotarget-07-4860-s001.pdf]

SUPPLEMENTARY FIGURES

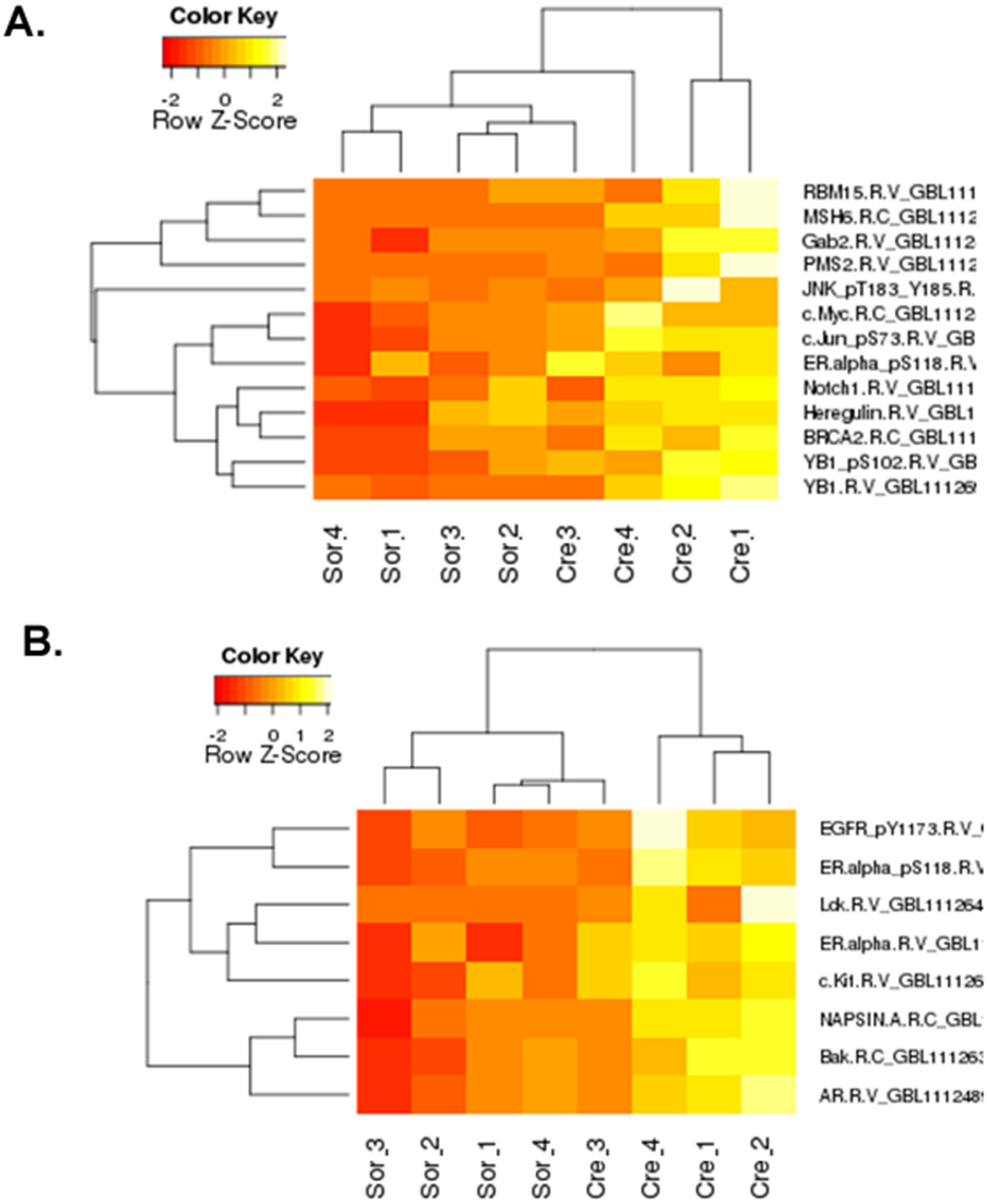

Supplementary Figure S1: RPPA analysis of signaling changes in mice induced by short-term Sorafenib treatment during PHx. Mdr2-KO mice were subjected to PHx at three months of age and treated with Sorafenib or Cremophor immediately and two hours following surgery. Total hepatic proteins from short-term Sorafenib or Cremophor treated mice were analyzed using RPPA. Heatmap dendrograms of selected proteins in mice at four hours **A.** and four days **B.** post PHx. Four mice from each group were subjected to RPPA analysis with 166 antibodies. Proteins exhibiting significant differences among the groups are presented. $P < 0.05$.

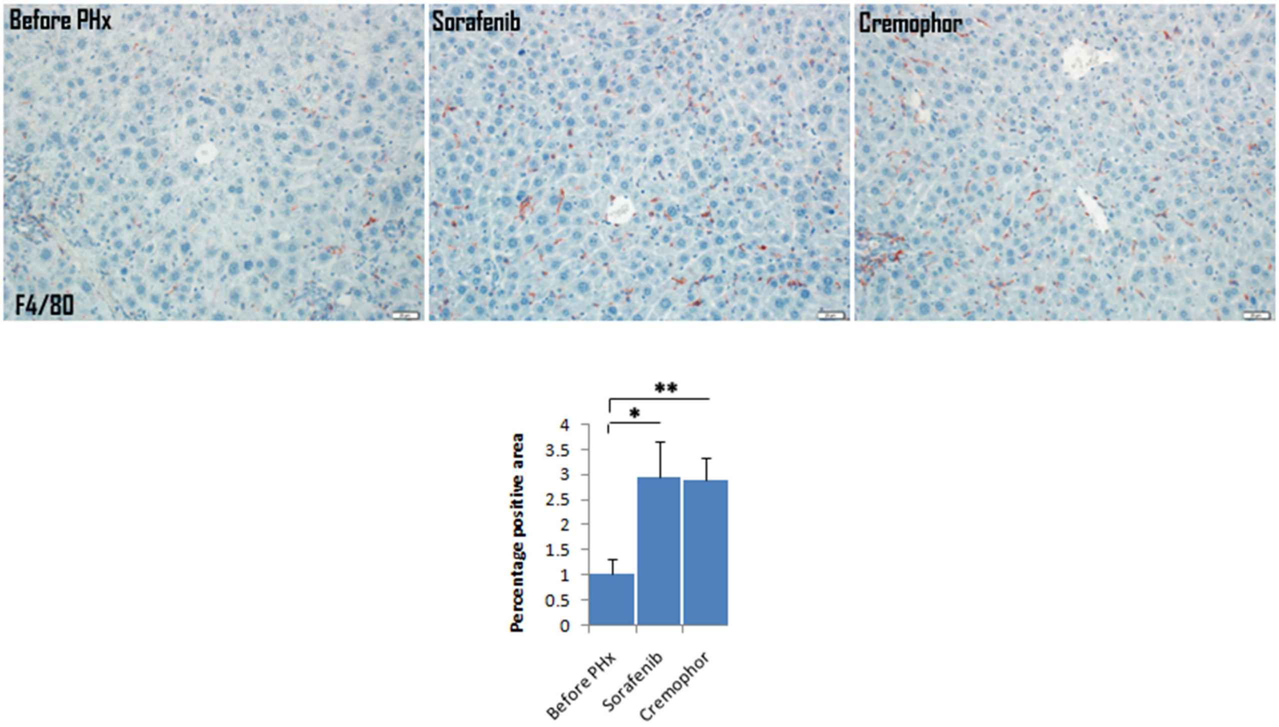

Supplementary Figure S2: F4/80 IHC staining for macrophages in liver tissues of short-term Sorafenib or Cremophor treated three- month-old before and four hours following PHx, quantified by the Ariol Slide Imaging System (an automated computerized slide imaging information collector) and analyzed by software (n = 6-7/group; **P < 0.01; *P < 0.05).
